# Cardiomyocytic FoxP3 is involved in Parkin-mediated mitophagy during cardiac remodeling and the regulatory role of triptolide

**DOI:** 10.7150/thno.71102

**Published:** 2022-02-28

**Authors:** Xi-Chun Pan, Ya-Lan Xiong, Jia-Hui Hong, Ya Liu, Yan-Yan Cen, Tao Liu, Qun-Fang Yang, Hui Tao, Yu-Nong Li, Hai-Gang Zhang

**Affiliations:** Department of Pharmacology, College of Pharmacy, Army Medical University, Chongqing 400038, China

**Keywords:** cardiac remodeling, mitophagy, Parkin, FoxP3, triptolide

## Abstract

**Rationale:** Forkhead/winged helix transcriptional factor P3 (FoxP3) is a well-studied transcription factor that maintains the activity of T cells, but whether cardiomyocytic FoxP3 participates in cardiac remodeling (CR) remains unclear. The present study was to investigate the role of cardiomyocytic FoxP3 in CR from the perspective of mitophagy.

**Methods:** CR was induced by angiotensin II (AngII) *in vitro*, or by isoproterenol (Iso) *in vivo* using male C57 mice or FoxP3^DTR^ mice. Histological changes were observed by hematoxylin-eosin and Masson staining. Molecular changes were detected by immunohistochemistry, immunofluorescence, immunoblotting, and real-time PCR. Mitophagy was shaped by transmission electron microscopy and co-localization. The mRNA expression was operated by siRNA or adeno associated virus (AAV). Molecular interactions were detected by co-localization, immunoprecipitation (IP), and chromatin IP.

**Results:** The expression and nuclear translocation of cardiomyocytic FoxP3 were downregulated in CR, while they were upregulated after triptolide (TP) treatment. In left ventricle (LV) remodeling in mice, autophagy was activated continuously in the myocardium, and TP significantly attenuated it. AngII induced massive mitophagy characterized by the activation of autophagy regulatory protein 5 (Atg5)-dependent autophagic flux. Critically, Parkin was identified as the main adaptor mediated myocardial mitophagy and was responsible for the effect of TP. Moreover, FoxP3 was responsible for the downregulation of Parkin and inhibited AngII-induced cardiac mitophagy. We found that mitophagy increased significantly and the inhibition of TP treatment reversed completely in FoxP3-deficient LVs. Mechanistically, FoxP3 interacted with a motif located downstream of the activating transcription 4 (ATF4)-binding motif involved in the promoter of *Parkin* and hijacked free nuclear ATF4 to decrease *Parkin* mRNA expression in CR.

**Conclusion:** Cardiomyocytic FoxP3 could negatively regulate Parkin-mediated mitophagy in CR, and restoring cardiomyocytic FoxP3 activity provided a cardioprotective strategy by inhibiting excessive cardiac mitophagy.

## Introduction

Cardiac remodeling (CR) is a complicated pathological process of molecular, cellular and interstitial changes that manifests clinically as alterations in the size, mass, geometry and function of the heart resulting from injury 1. Hemodynamic overloading, neurohormonal factors, myocarditis, myocardial infarction (MI), and other factors are responsible for CR 1. CR is involved in almost all types of cardiovascular diseases and is considered a major therapeutic goal in the treatment of patients with hypertension, cardiomyopathy, and chronic heart failure.

Macroautophagy (autophagy) is a conserved process for the bulk degradation and recycling of damaged organelles 2. Controlled by autophagy-related proteins (Atgs) and microtubule-associated protein 1 light chain 3 (LC3), the autophagy machinery engulfs cargos via double membrane-surrounded autophagic vesicles (autophagosomes), which culminates in lysosomal degradation through the fusion with lysosomes (autolysosomes) 2. Autophagy plays an essential role in CR to maintain cardiac function and cellular homeostasis or to mediate cell death in the heart 3, 4. Mitochondrial damage is a major intracellular change and is considered cytotoxic to cardiomyocytes, the main cell type of the heart 4. Mitophagy targeting damaged mitochondria has been reported to exacerbate the propensity for spontaneous CR in laboratory animals and is suggested to be a potential target for reversing CR 3. To our knowledge, mitophagy employs autophagy receptors or adaptors to recognize damaged mitochondria 5. Several autophagy receptors, such as BCL2 interacting protein 3 (BNIP3) and BNIP3-like protein (NIX), and autophagy adaptors, such as PTEN-induced kinase 1 (Pink1) and Parkin, have been shown to be involved in cardiac mitophagy 3, 6. However, the detailed upstream regulatory mechanisms of mitophagy in CR are still not well recognized.

Forkhead/winged helix transcriptional factor P3 (FoxP3) is a well-studied transcription factor that maintains the activity of regulatory T cells (Tregs). In a mouse MI model, Foxp3 in Tregs suppressed the activation and migration of immune cells within the myocardium and interstitium, attenuated cardiac inflammation, and thus ameliorated myocardial damage and subsequent CR 7. Adoptive transfer of Tregs inhibited CR in AngII-induced hypertensive mice and showed a protective effect against aldosterone-induced vascular injury 8, 9. However, less has been reported about the role of cardiomyocytic FoxP3 in CR. Previously, we reported for the first time that cardiomyocytic FoxP3 was downregulated in CR, while triptolide (TP) upregulated FoxP3 expression and attenuated CR, suggesting that FoxP3 might participate in part in CR 10. Based on a series of pre-experiments, we hypothesized that FoxP3 might play a negative role in myocardial mitophagy in CR. In this study, the role of cardiomyocytic FoxP3 and its regulatory mechanism in CR were further investigated combined with insight into the regulation of cardiac mitophagy through the use of TP as a FoxP3 activator.

## Materials and methods

### Animals and treatments

Male wild-type C57 mice (8-10 weeks old, weighing 20-25 g) were supplied by the Experimental Animal Center of our university. The animal experimental protocol was reviewed and approved by the Ethics Committee for Animal Experimentation of Army Medical University and was performed according to the Guide for the Care and Use of Laboratory Animals from the National Institutes of Health (8th Ed., 2011). CR in mice was induced by continuous infusion of Iso (40 mg/kg/day; s.c.; Sigma, St. Louis, MO, USA) for 14 days with or without TP (10, 30, or 100 μg/kg; i.p.; Beijing Medicass Biotech., Beijing, China) as described previously 10. Moreover, temporal CR changes in mice were also observed by administration of Iso (40 mg/kg/day; s.c.) for 0, 1, 3, 7, or 14 days with or without TP (100 μg/kg; i.p.). Male mice harboring the FoxP3-diphtheria toxin receptor genotype (FoxP3^DTR^) were obtained from Prof. Li-Lin Ye of our university. To eliminate FoxP3-positive cardiomyocytes, mice received an injection of diphtheria toxin (50 μg/kg; i.p.; Sigma) before CR induction. Then, FoxP3^DTR^ or wild-type C57 mice were given with continuous infusion of Iso (40 mg/kg/day; s.c.) with or without TP (100 μg/kg; i.p.) for 14 days. At the end of animal experiments, the mid-ventricle was stored in liquid nitrogen for immunoblotting (IB), fixed with a neutral formalin solution and embedded in paraffin for histological assays, or fixed with a glutaraldehyde solution (2.5% glutaraldehyde dissolved in 0.1 M PBS; m/v; pH 7.4) for transmission electron microscopy (TEM) observations.

### Histological observations

Paraffin sections (5 μm thick) from mouse ventricular samples were used for subsequent hematoxylin-eosin (HE) staining, Masson's trichrome staining, and immunohistochemistry (IHC) assays that were probed with anti-FoxP3 (Santa Cruz, CA, USA), as previously reported 11. The histological pictures were scanned using a light microscope and analyzed by ImageJ (http://rsb.info.nih.gov/ij/). The inflammation score indicated inflammatory cell infiltration was calculated according to previous report 12. For TEM observations, glutaraldehyde-fixed samples were postfixed, dehydrated, rinsed and finally embedded in an araldite mixture. Sections were then observed with a TEM system (JEM-1400PLUS; JEOL, Tokyo, Japan).

### Cell cultures

The H9c2 cardiomyocyte cell line was purchased from ATCC (Manassas, VA, USA) and cultured in DMEM (Grand Island, NY, USA) supplemented with 10% (v/v) fetal bovine serum (FBS; Gibco, NY, USA) at 37 °C in an incubator with 5% CO_2_. Neonatal rat ventricular cardiomyocytes (NRVMs) were isolated from 1- to 2-day-old SD rats using an enzymolytic method as described previously 13 and cultured in DMEM supplemented with 10% (v/v) FBS and 0.1 mM 5-bromo-2-deoxyuridine after preparation. For both H9c2 cells and NRVMs, the culture medium was replaced with serum-free medium overnight before treatment, and then angiotensin II (AngII; 1 μM; Sigma) was used to stimulate cell hypertrophy and mitophagy *in vitro* with or without TP (10 μg/L) as indicated for 4 h.

### Real-time PCR

H9c2 cells or NRVMs were inoculated in 12-well plates (5 × 10^4^ cells/well) and treated with AngII (1 μM) with or without TP (10 μg/L) as indicated. After treatment, the cells were harvested using TRIzol reagent (Takara, Dalian, Liaoning, China) to extract total RNA. The amounts of 1 μg of total RNA from each group were reverse-transcribed into cDNA using the PrimeScript^TM^ RT Reagent Kit (Takara). The mRNA expression levels were determined by real-time PCR using specific primer pairs supplied by Sangon Biotech (Shanghai, China; Table 1). Real-time PCR was performed using SsoAdvanced SYBR Green Supermix (Bio-Rad, CA, USA) with a CFX PCR system (Bio-Rad) under the following procedure: 40 cycles each containing 95 °C for 10 s and 55 °C for 30 s. Data were calculated using the 2^-△△Ct^ method by normalization to β-actin 14.

### Immunofluorescence

For tissue immunofluorescence (IF), paraffin sections of mouse ventricles were used, and the procedure was performed as previously reported 15. Briefly, α-actinin or vimentin was probed with the corresponding mouse antibody (Santa Cruz; dilution 1:200) overnight at 4 °C, followed by Alexa Fluor 555 (AF555)-conjugated secondary antibody (Thermo Fisher, Waltham, MA, USA; 1:400) for 2 h at 4 °C. Then, the same sections were probed with rabbit anti-LC3 (Cell Signaling; Beverly, MA, USA) followed by AF488-conjugated secondary antibody (Thermo Fisher) under the same conditions described above. The nuclei were stained with 5 μg/mL DAPI for 5 min. Slides were observed using a Zeiss LSM800 confocal laser microscope (Zeiss, Jena, Germany), and the data were analyzed using ImageJ software.

For IF staining, H9c2 cells or NRVMs were inoculated on glass slides in 24-well plates (2 × 10^4^/well). To detect FoxP3 expression, cells were treated with AngII (1 μM) with or without TP (10 μg/L) for 1 h, fixed with 4% paraformaldehyde in PBS for 15 min, blocked with 1% BSA in PBS supplemented with 0.1% Triton X-100, and probed with anti-FoxP3 antibody (Santa Cruz; 1: 200) overnight at 4 °C, followed by the application of AF488-conjugated secondary antibody (Thermo Fisher; 1: 400) for 2 h at 4 °C. The nuclei were stained by DAPI (5 μg/mL) for 5 min, and the results were observed and analyzed as shown above. To detect LC3 dots, cells were treated as described for FoxP3 for 4 h and probed with an anti-LC3 antibody (Cell Signaling; 1:200) followed by an AF488-conjugated secondary antibody. To detect autophagic flux, H9c2 cells were transfected using an RFP-GFP-LC3 kit (Thermo Fisher) according to the user manual and treated as described for FoxP3 for 4 h. Autophagosomes (yellow) and autolysosomes (red) in live cells were observed immediately.

### Colocalization

To detect mitophagy, H9c2 and NRVMs grown on glass slides in 24-well plates (2 × 10^4^ cells/well) were treated with AngII (1 μM) with or without TP (10 μg/L) for 4 h. Mitochondria were probed with MitoTracker red reagent (0.1 μM; Thermo Fisher) for 20 min before IF staining. Then, the cells were fixed, blocked, and probed with anti-LC3 followed by AF488-conjugated secondary antibody as described above. In addition, Parkin-mediated mitophagy was detected by staining with MitoTracker red, mouse anti-Parkin (Abcam, Waltham, MA, USA; 1:200) followed by AF647-conjugated secondary antibody (Thermo Fisher; 1:400), and rabbit anti-LC3 followed by AF488-conjugated secondary antibody. To detect Pink1-LC3 and Parkin-LC3 colocalization, Pink1 or Parkin was probed with the corresponding mouse antibodies (Abcam; 1:200) followed by AF555-conjugated secondary antibody (Thermo Fisher; 1:400) and rabbit anti-LC3-conjugated secondary antibody and then AF488-conjugated secondary antibody. The nuclei were stained by DAPI (5 μg/mL) for 5 min. The colocalization of mitochondria (red), LC3 (green), and Pink1 or Parkin (shown in light blue) was observed using confocal imaging, and Pearson's correlation was calculated using ImageJ.

To detect activating transcription 4 (ATF4)-FoxP3 colocalization, H9c2 cells and NRVMs were treated as described for FoxP3 IF staining. FoxP3 was probed with mouse anti-FoxP3 followed by AF488-conjugated secondary antibody (green), and ATF4 was probed with rabbit anti-ATF4 (Cell Signaling; 1:200) followed by AF555-conjugated secondary antibody (1:400; red).

### Immunoblotting and immunoprecipitation

Total lysates from cultured cells or hearts were extracted using Cell Lysis Buffer for Western blotting and IP (Beyotime, Wuhan, Hubei, China). Cytoplasmic and nuclear lysates were extracted using a Nuclei Extraction Kit (Beyotime). Mitochondrial lysates were extracted using a Cell Mitochondria Isolation Kit (Beyotime). For immunoblotting (IB), 20 μg of protein samples was boiled for 5 min and separated using SDS-PAGE. The protein bands were then transferred onto polyvinylidene fluoride (PVDF) membranes, blocked with 5% skim milk for 2 h at 37 °C, and probed with the designated primary antibodies (dilution 1:1000) overnight at 4 °C, followed by corresponding horseradish peroxidase (HRP)-conjugated secondary antibodies (Thermo Fisher; 1:10000) at 37 °C for 2 h. Bands were then developed with the SuperSignal chemiluminescent substrate (Bio-Rad) under a ChemiDoc™ Touch Imaging System (Bio-Rad) and analyzed using ImageLab software (Bio-Rad). Primary antibodies against FoxP3, ATF4, LC3, Atg5 (Cell Signaling), Pink1, Parkin, Histone H3 (Beyotime), CoxIV, and GAPDH (Beyotime) were used herein.

Immunoprecipitation (IP) experiments were performed using Protein A/G magnetic beads (Thermo Fisher) according to the user manual. Briefly, cytosolic or nuclear lysates (0.5 mg) were incubated with the indicated primary antibodies (5 μg) at 4 °C overnight. Then, the immune complexes were collected using Protein A/G beads and subjected to IB assessment.

### Knockdown and overexpression of *FoxP3*

*FoxP3* knockdown was performed using a specific siRNA with a control siRNA synthesized by Sangon Biotech (Table 1). The transfection procedure was carried out using the lipidosome method with Lipofectamine 2000 reagent (Thermo Fisher) according to the user manual. In brief, H9c2 cells were cultured in 12-well plates (3 × 10^4^ cells/well) until they reached 60% confluence and then transfected with the assembled lipidosome-containing siRNA for 8 h. Subsequently, the supernatants were replaced with complete DMEM with 10% FBS. After 16 h of recovery, the cells were used for further treatment.

*FoxP3* overexpression was performed using an adeno associated virus harboring rat *FoxP3* (FoxP3-AAV; Sangon). Briefly, H9c2 cells were cultured in 12-well plates to 80% confluence (5 × 10^4^ cells/well) and then transfected with FoxP3-AAV (1 × 10^7^ IU/well) for 16 h. The supernatants were replaced with DMEM containing 10% FBS for a 16-h renewed culture, and the cells were then used for further treatment.

### Chromatin immunoprecipitation

Chromatin immunoprecipitation (ChIP) was performed according to the user manual provided with the Agarose ChIP kit (Pierce, Rockford, IL, USA). In brief, after cross-linking with formalin solution supplemented with glycine, cell chromatin was fragmented using micrococcal nuclease. The supernatant containing digested chromatin was collected and used for pull-down by a ChIP-grade anti-FoxP3 antibody (Santa Cruz). ChIP products were amplified by real-time PCR (ChIP-qPCR) with ChIP primers specific to the promoter of the *Parkin* gene (Sangon; Table 1); additionally, the input samples were analyzed using control primers specific to the *gapdh* promoter involved in the kit. Data were calculated using the 2^-△△Ct^ method as described above.

### Statistics

All data are expressed as the mean ± SEM of at least 3 independent experiments. The statistical significance of differences between groups was determined by one-way ANOVA using SPSS 11.0. Differences with a *P* value less than 0.05 were considered statistically significant.

## Results

### The expression and nuclear translocation of cardiomyocytic FoxP3 were downregulated in CR

**Figure 1:**
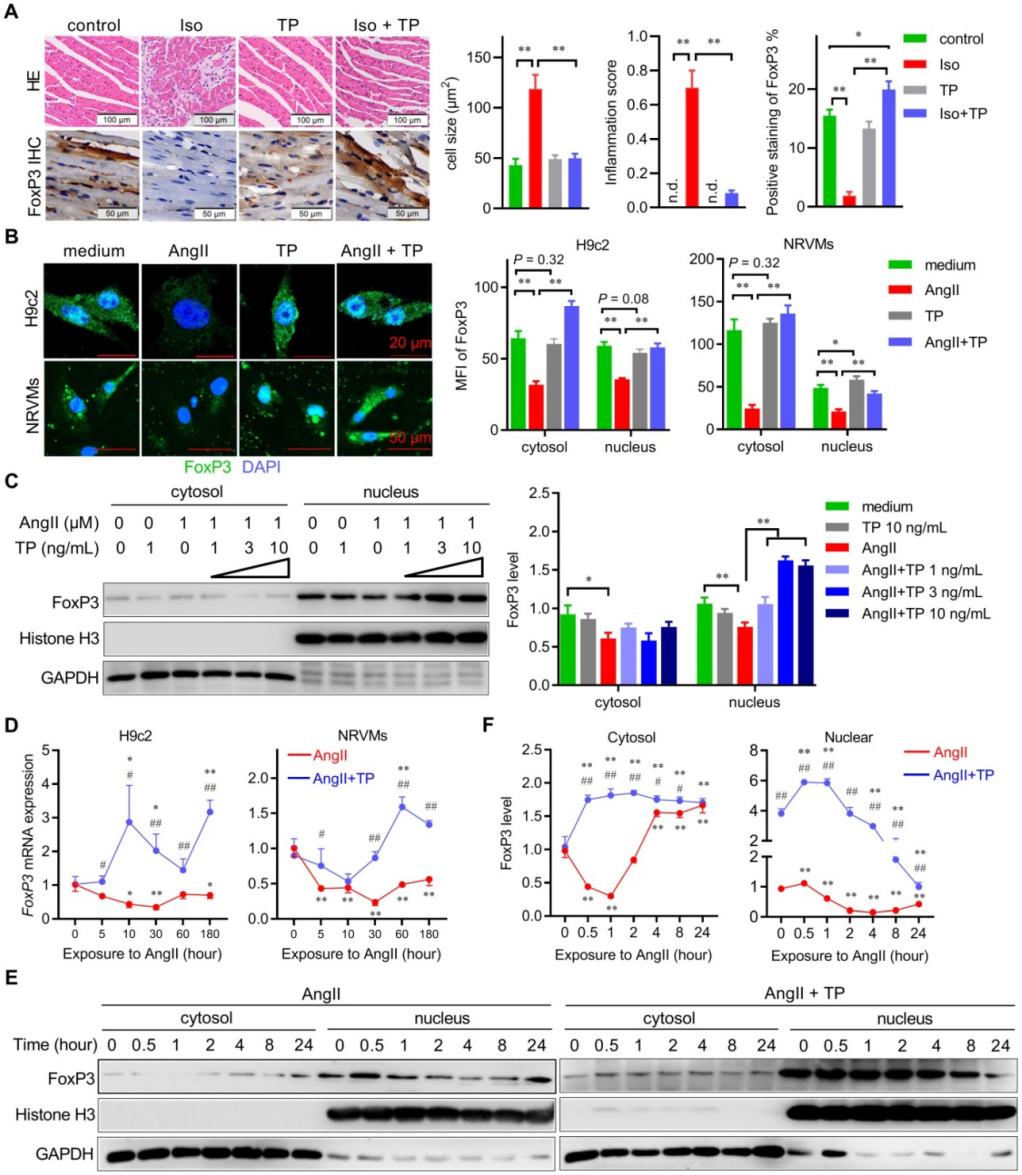
** The expression and nuclear translocation of cardiomyocytic FoxP3 are downregulated in cardiac remodeling (CR)**. (A) CR was induced by continuous Iso infusion (40 mg/kg/day; s.c.) with or without TP (100 μg/kg; i.p.) for 14 days in mice. Cross-sections of middle ventricles were used for hematoxylin-eosin (HE) staining and immunohistochemistry (IHC) assays of FoxP3. Histograms represent cell size, inflammation score, and percent positive staining of FoxP3 (n = 3). "n.d." indicates non-detected, ^*^P < 0.05, ^**^P < 0.01. (B) H9c2 cells and NRVMs grown on glass slides were treated with AngII (1 μM) with or without TP (10 μg/L) for 1 h and then fixed, blocked, and probed with anti-FoxP3 antibody (green). Histograms indicate the mean fluorescence index (MFI) within cells (n = 20). ^*^P < 0.05, ^**^P < 0.01. (C) H9c2 cells grown in 10-cm dishes were treated with AngII (1 μM) with or without TP (1, 3, 10 μg/L) for 1 h, and then cytoplasmic and nuclear lysates were subjected to immunoblotting (IB) using FoxP3 antibody. Histograms represent protein ratios normalized to GAPDH (cytosol) or histone H3 (nucleus; n = 3). (A-C) ^*^P < 0.05, ^**^P < 0.01. (D) H9c2 cells and NRVMs grown in 12-well plates were treated with AngII (1 μM) with or without TP (10 μg/L) for up to 3 h, and then cells were harvested for real-time PCR analysis of *FoxP3* mRNA expression by normalization to β-actin (n = 3). (E) H9c2 cells and NRVMs grown in 10-cm dishes were treated with AngII (1 μM) with or without TP (10 μg/L) for up to 24 h, and then cells were harvested for IB of FoxP3 as shown above. (D-E) ^*^P < 0.05, ^**^P < 0.01 vs. 0 h; ^#^*P* < 0.05, ^##^*P* < 0.01 vs. AngII.

A previous study in our laboratory suggested that FoxP3 was expressed in cardiomyocytes and downregulated in hypertrophic cardiomyocytes 10. Herein, we reproduced the *in vivo* experiments using paraffin sections of remodeled left ventricles (LVs) of mice induced by Iso with or without the presence of TP, a previously reported cardiac protective agent 10, 11. The results showed that Iso induced remarkable LV remodeling, including hypertrophy of cardiomyocytes, myocardial fiber disruption, focal necrosis, and interstitial fibrosis and inflammatory cell infiltration, compared with the control, while TP treatment markedly decreased LV remodeling including cardiac fibrosis and inflammation score compared with the Iso group (Figure 1A). Consistently, IHC results from this study showed that cardiomyocytic FoxP3 in remodeled LVs induced by Iso was markedly decreased, while it was restored in line with the amelioration of LV remodeling by TP treatment (Figure 1A). However, TP alone could not alter FoxP3 expression.

Subsequently, we further investigated the spatiotemporal expression pattern of FoxP3 in CR *in vitro*. First, IF staining showed that the cytosol and nuclear expression of FoxP3 (green) were significantly downregulated in cardiomyocytes (H9c2 & NRVMs) treated with AngII for 1 h; however, they were upregulated significantly by TP treatment compared with AngII alone (Figure 1B). Similarly, TP treatment (1 h) increased the level of nuclear FoxP3 compared with AngII alone in a dose-dependent manner (Figure 1C). Second, the temporal changes in *FoxP3* expression in CR *in vitro* were determined. Real-time PCR results showed that *FoxP3* mRNA expression in both H9c2 cells and NRVMs was downregulated rapidly with AngII stimulation, while FoxP3 mRNA expression was upregulated by TP treatment (Figure 1D). Furthermore, the level of nuclear FoxP3 continuously decreased after AngII treatment from 1 h to 24 h. However, the level of cytosolic FoxP3 was only decreased by AngII for up to 2 h and then increased (Figure 1E-F). Similar to the mRNA level data, TP treatment significantly increased the levels of both cytosolic and nuclear FoxP3 compared with AngII alone (Figure 1E-F). Together, these data indicated that the expression and nuclear translocation of FoxP3 were downregulated by AngII, but these changes were reversed by TP treatment, which further suggested that TP could restore the transcriptional activity of FoxP3. Notably, the target gene of cardiomyocytic FoxP3 in CR remains unclear.

### Autophagy was extensively activated in the early stage of CR

**Figure 2:**
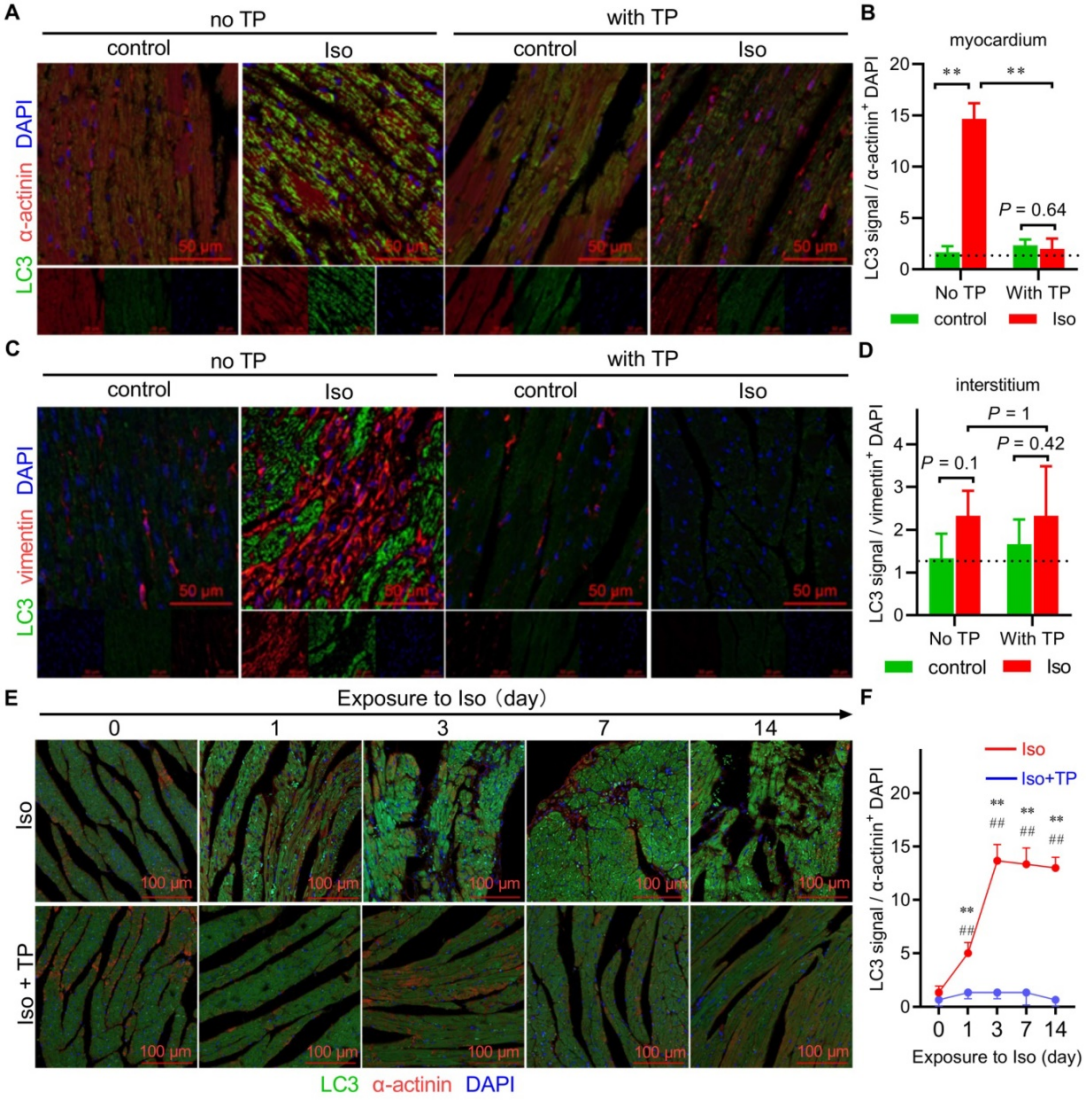
** Autophagy is extensively activated in the early stage of cardiac remodeling (CR).** (A-D) Ventricular sections were prepared as shown in Figure 1A and were used for immunofluorescence (IF) staining with antibodies against α-actinin or vimentin (red) plus LC3 (green). Slides were observed under a confocal laser microscope, and the data were analyzed by ImageJ (n = 3).^ **^P < 0.01. (E-F) Mice were treated with Iso (40 mg/kg/day; s.c.) for 0, 1, 3, 7, or 14 days with or without TP (100 µg/kg; i.p.). Subsequent IF experiments were performed as described above using antibodies against α-actinin (red) and LC3 (green). n = 3; ^**^P < 0.01 vs. 0 day; ^##^*P* < 0.01 vs. Iso.

Autophagy plays a crucial role in the pathophysiological process of CR by maintaining cellular homeostasis and participating in cell death 16. Therefore, we hypothesized that FoxP3 might be involved in autophagy regulation. Herein, experiments were initiated with the identification of cell types that undergo autophagy in CR. Cardiomyocytes and fibroblasts are the main structural cell types in the heart; therefore, immunofluorescent colocalization of LC3 (an indicator of autophagy) with α-actinin used as a marker of cardiomyocytes or vimentin as a marker of fibroblasts was performed using paraffin sections as mentioned above. We found that massive LC3 puncta (green dots) occurred in cardiomyocytes but not in fibroblasts in the remodeled LV of mice in the Iso group, indicating that autophagy was activated in cardiomyocytes in CR (Figure 2A-D). Similar to its anti-remodeling effect, TP showed a strong inhibition of autophagy.

The above data were produced using remodeled ventricles. Herein, we further detected autophagic alterations in the overall process of CR using a series of remodeling ventricle samples treated with Iso at different time points (0 days, 1 day, 3 days, 7 days, and 14 days). The results showed that LC3 puncta accumulated from the 1st day and were continuously activated up to the 14th day during remodeling (Figure 2E-F). Not surprisingly, TP also continuously exhibited the anti-autophagy effect. Taken together, these data suggested that autophagy occurred early and was continuously activated within cardiomyocytes in CR, and autophagy inhibition might be beneficial to protect the heart against the stimulation of neurohormonal factors such as Iso.

### Massive Atg5-dependent mitophagy in CR

**Figure 3:**
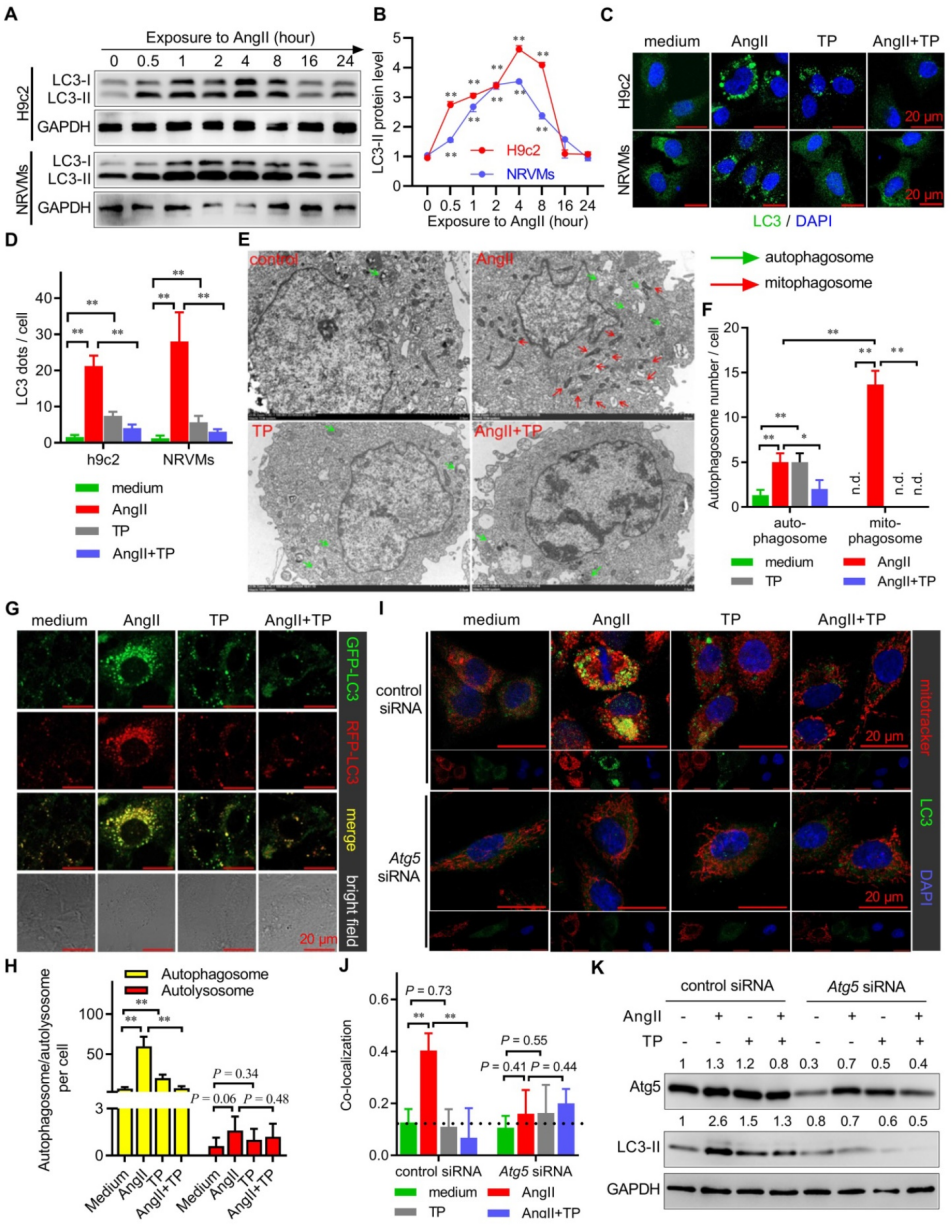
** Massive Atg5-dependent mitophagy in cardiac remodeling (CR).** (A-B) H9c2 cells and NRVMs were treated with AngII (1 μM) for up to 24 h. The levels of LC3-I/II were determined by immunoblotting (IB) and normalized to GAPDH (n = 3). (C-D) H9c2 cells and NRVMs grown on glass slides were treated with AngII (1 μM) with or without TP (10 μg/L) for 4 h and then probed with anti-LC3 antibody (green) and DAPI (blue). LC3 dots were observed using confocal microscopy and counted in ImageJ (n = 20). (E-F) H9c2 cells were treated as described in C and then collected for transmission electron microscopy (TEM) observations (×3000). (G-H) H9c2 cells were transfected with an RFP-GFP-LC3 kit and treated as described in C. Autophagosomes (yellow) and autolysosomes (red) were observed and counted as shown in C (n = 20). (I-J) H9c2 cells were transfected with control and *Atg5* siRNAs. Cells were then treated as shown in C, and mitochondria were probed with MitoTracker red (red) for 20 min before fixation. Fixed cells were probed with anti-LC3 antibody (green). Pearson's correlation indicating mitochondria-LC3 colocalization (yellow) was calculated using ImageJ (n = 20). (K) H9c2 cells were treated as shown in I and detected as shown in A. The values shown above the bands are the mean protein ratios normalized to GAPDH (n = 3). "n.d." indicates non-detected, ^*^P < 0.05, ^**^P < 0.01.

To further investigate the characteristics of autophagy in CR, several *in vitro* experiments were performed in this study using cardiomyocytes (NRVMs and H9c2) treated with AngII. First, we detected whether and when autophagy occurred in cardiomyocytes after treatment with AngII. The IB results showed that the LC3-II level increased from 0.5 h to 8 h and peaked at 4 h in both the H9c2 cell line and primary cardiomyocytes (NRVMs), which were treated with AngII for up to 24 h (Figure 3A-B). Therefore, 4 h was selected as the checkpoint for observing autophagic alterations of cardiomyocytes. Moreover, IF imaging showed that AngII induced significant LC3 puncta accumulation in both H9c2 cells and NRVMs; however, these puncta were decreased by TP treatment (Figure 3C-D). These results supported the conclusion that autophagy was activated by AngII but was decreased by TP in cardiomyocytes.

Subsequently, we further detected the type of autophagy that occurred in CR by observing the morphological characteristics of autophagosomes using TEM. The results showed that AngII markedly increased the number of mitochondria-containing autophagosomes (mitophagosomes) and weakly increased the number of normal autophagosomes (Figure 3E-F). However, both mitophagosomes and normal autophagosomes induced by AngII were decreased by TP. These results clearly indicated that AngII mainly induced mitophagy in cardiomyocytes.

Given that both extreme activation and degradation blockade contributed to autophagosome accumulation, AAV harboring pH-sensitive GFP-RFP-LC3 was employed to detect which process was targeted by AngII and TP. Our results showed that the number of autophagosomes (yellow dots), not autolysosomes (red dots), was remarkably increased by AngII and decreased by TP, suggesting that AngII and TP acted on autophagic activation but not degradation (Figure 3G-H).

Moreover, classic mitophagy is Atg5-Atg12-Atg16L1 dependent because this system is critical for LC3 conjugation 6. Using a specific siRNA targeting *Atg5* mRNA (*Atg5* siRNA), we found that AngII could not induce mitophagy (yellow dots) in H9c2 cells with* Atg5* knockdown, in contrary to H9c2 cells treated with control siRNA (Figure 3I-J). Consistently, TP also lost the ability to inhibit mitophagy in H9c2 cells with* Atg5* knockdown. Similarly, IB results also showed that AngII and TP lost their abilities to modulate LC3-II expression (Figure 3K). These results indicated that mitophagy activation in CR was Atg5-dependent and further suggested that TP indeed acted on the autophagic activation process.

### CR induced mitophagy via a Pink1-Parkin-LC3 pathway

**Figure 4:**
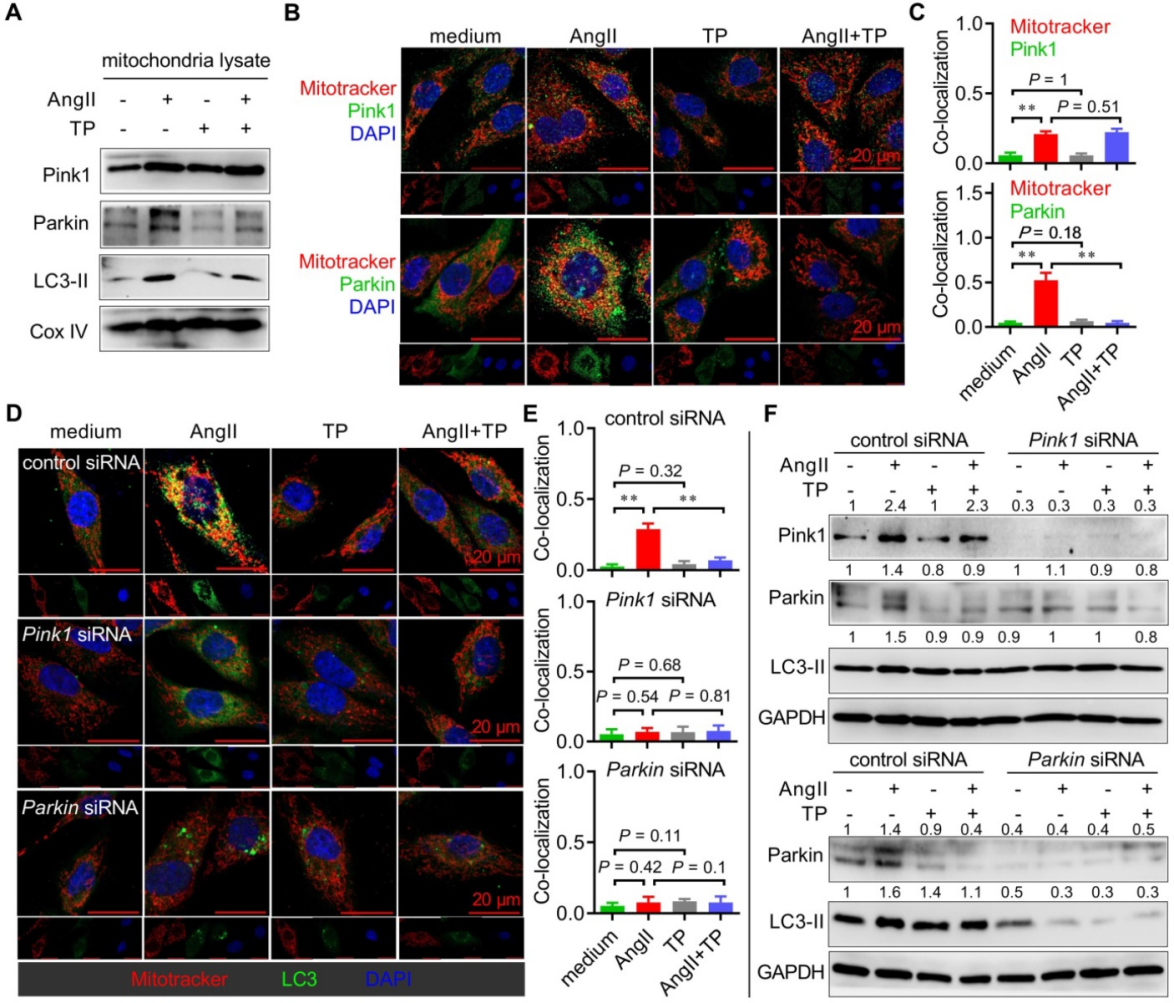
** Cardiac remodeling (CR) induces mitophagy via a Pink1-Parkin-LC3 pathway.** (A) NRVMs grown in 10-cm dishes were treated as shown in Figure 3C, and mitochondrial lysates were detected by immunoblotting (IB) using antibodies against Pink1, Parkin, LC3 and CoxIV (internal control). (B-C) H9c2 cells grown on slides were treated as shown in Figure 3C, and mitochondria (red) were probed as shown in Figure 3I. Cells were then probed with anti-Pink1 or anti-Parkin antibody (green). Pearson's correlation indicating colocalization of mitochondria with Pink1 or Parkin was calculated as shown in Figure 3I. (D-E) H9c2 cells were transfected with control siRNA, *Pink1* siRNA and *Parkin* siRNA. Experiments were carried out as shown in Figure 3I. (F) H9c2 cells were treated as shown in D, and the total lysates were detected by IB using antibodies against Pink1, Parkin, LC3 and GAPDH (internal control). The values shown above the bands are the mean protein ratios normalized to GAPDH (n = 3). ^**^P < 0.01.

The recruitment of autophagic machinery by mitochondria is mediated by some LC3 receptors, such as BNIP3 and NIX, as well as LC3 adaptors, such as the Pink1-Parkin axis and optineurin 3. Therefore, extracts from mitochondria of NRVMs were detected by IB with antibodies against these LC3 receptors and adaptors. Although the protein levels of Pink1, Parkin, and LC3-II were dramatically increased in the AngII group, only those of Parkin and LC3-II were decreased by TP treatment (Figure 4A). Moreover, we also performed colocalization experiments to observe whether Pink1 and Parkin were recruited by mitochondria in H9c2 cells induced by AngII. Consistently, colocalization of Pink1 or Parkin (green) with mitochondria (red) was increased in the AngII group; however, only Parkin-mitochondria colocalization was inhibited by TP treatment (Figure 4B-C). These results suggested that AngII induced cardiac mitophagy by the Pink1-Parkin pathway and that TP exhibited anti-mitophagy activity by downregulating the expression of Parkin.

To further verify the above findings, siRNA against *Pink1* mRNA or *Parkin* mRNA was used to determine the role of Pink1 and Parkin in cardiac mitophagy *in vitro*. First, colocalization experiments were again performed, and the results showed that AngII-induced mitophagy (yellow dots) persisted in H9c2 cells treated with control siRNA, while it was lost in H9c2 cells with *Pink1* or *Parkin* knockdown (Figure 4D-E). Moreover, TP lost the ability to inhibit mitophagy in H9c2 cells with *Pink1* or *Parkin* knockdown. Similarly, the IB results showed that AngII and TP lost their abilities to modulate the expression of Parkin and LC3-II in H9c2 cells with *Pink1* knockdown (Figure 4F, upper panel), as well as on the expression of LC3-II in H9c2 cells with *Parkin* knockdown (Figure 4F, lower panel). Importantly, the LC3-II level was dramatically decreased in H9c2 cells with *Parkin* knockdown, whereas it showed no notable decrease in H9c2 cells with *Pink1* knockdown. Together, our data indicated that AngII induced mitophagy through the Pink1-Parkin pathway and that Parkin seemed more critical for the activity of AngII and TP.

### FoxP3 knockdown promoted Parkin-mitophagy in cardiomyocytes

**Figure 5:**
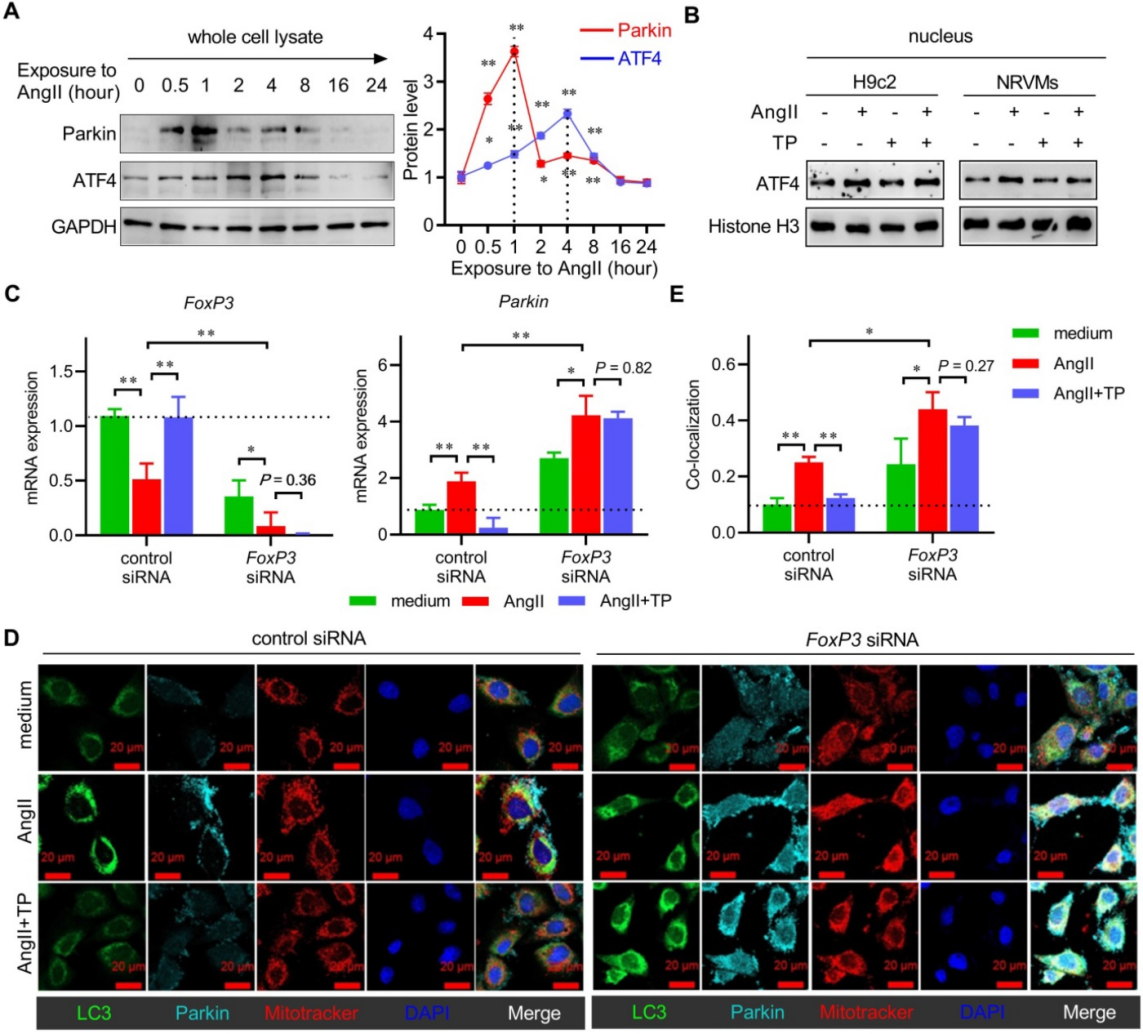
** Knocking down FoxP3 promotes Parkin-mediated mitophagy in cardiomyocytes.** (A) NRVMs were treated, and the levels of Parkin and ATF4 were determined as shown in Figure 3A (n = 3). (B) H9c2 cells and NRVMs were treated with AngII (1 μM) with or without TP (10 μg/L) for 4 h. Nuclear lysates were extracted for immunoblotting (IB) using antibodies against ATF4 and histone H3 (internal control). (C) H9c2 cells grown in 12-well plates were transfected with control siRNA and *FoxP3* siRNA and then treated as shown in B for 15 min. *FoxP3* or *Parkin* mRNA expression was determined as shown in Figure 1D. (D-E) H9c2 cells grown on glass slides were transfected and treated as shown in C. Mitochondrial (red), LC3 (green) and Parkin (light blue) staining and colocalization (white) were performed as shown in Figure 3I. ^*^P < 0.05, ^**^P < 0.01.

The above results demonstrated that Parkin played a crucial role in cardiac mitophagy. To our knowledge, ATF4 has been reported to be a transcription factor that activates *Parkin* mRNA expression. Therefore, we observed the protein levels of Parkin and ATF4 in the total cell lysate from H9c2 cells treated with AngII for up to 24 h. The IB results showed that the protein level of Parkin in the AngII group increased rapidly and then decreased gradually along with mitophagy degradation, and the peak occurred at 1 h (Figure 5A). The expression pattern of ATF4 was consistent with that of Parkin; however, the peak occurred at 4 h, which lagged compared with that of Parkin. In addition, TP did not alter the nuclear level of ATF4 in either H9c2 cells or NRVMs (Figure 5B). The results suggested that an ATF4-independent regulatory mechanism was involved in the regulation of Parkin, which was responsible for AngII and TP.

Combining the results of Figure 1 and 4, the expression and nuclear translocation of FoxP3 showed an inverse relationship with Parkin in CR. Therefore, we hypothesized that it might play a negative role in cardiac mitophagy. Herein, we used FoxP3 knockdown H9c2 cells (using *FoxP3* siRNA) to investigate the role of FoxP3 in Parkin-mediated mitophagy. For mRNA expression, the results showed that downregulation effect of AngII on *FoxP3* was weakened and the upregulation effect of TP on *FoxP3* was completely lost in cells with *FoxP3* knockdown (Figure 5C). Importantly, FoxP3 knockdown significantly enhanced *Parkin* mRNA expression. We found that the upregulation effect of AngII on *Parkin* was weakened and the downregulation effect of TP on *Parkin* was completely lost in cells with *FoxP3* knockdown (Figure 5C).

For mitophagy activation, AngII could induce Parkin-mediated mitophagy (mitochondria-Parkin-LC3 colocalization) in cells without *FoxP3* knockdown but showed a stronger effect in cells with *FoxP3* knockdown (Figure 5D-E). Consistent with the mRNA level results mentioned above, we found that the ability of TP to inhibit Parkin-mediated mitophagy was lost in cells with *FoxP3* knockdown (Figure 5D-E). Together, these results suggested that FoxP3 inhibited Parkin-mediated cardiac mitophagy and was strictly required for the effect of TP.

### Overexpression of FoxP3 inhibited Parkin-mediated mitophagy in cardiomyocytes

**Figure 6:**
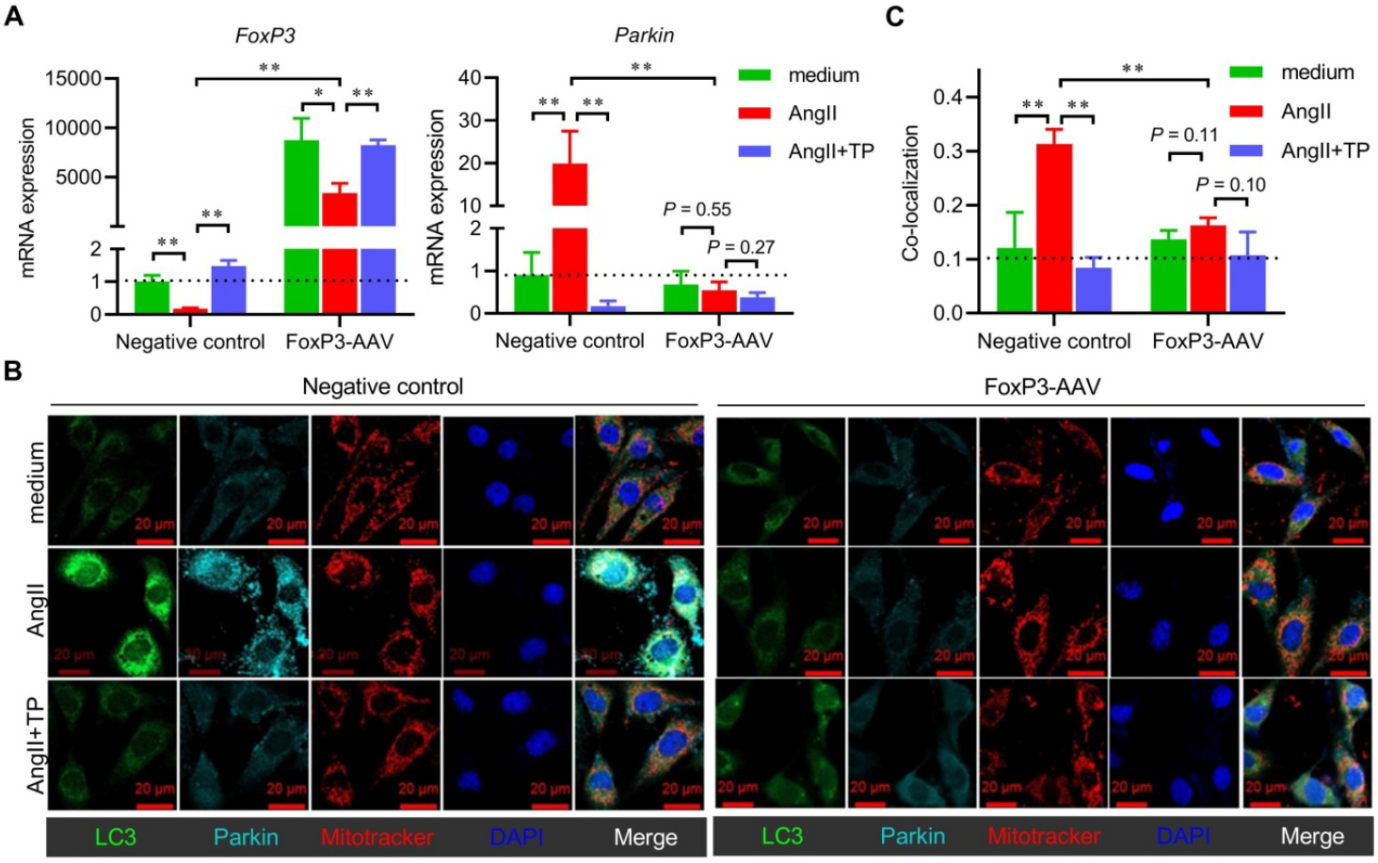
** Overexpression of FoxP3 inhibits Parkin-mediated mitophagy in cardiomyocytes.** (A) H9c2 cells grown in 12-well plates were transfected with negative control AAV and FoxP3 AAV and treated as shown in Figure 5C. *FoxP3* or *Parkin* mRNA expression was determined as shown in Figure 1D. (B-C) H9c2 cells grown on glass slides were transfected and treated as shown in A. Immunofluorescence (IF) staining and colocalization (white) calculations were performed as shown in A. ^*^P < 0.05, ^**^P < 0.01.

We also evaluated the role of FoxP3 in Parkin-mediated mitophagy in H9c2 cells with *FoxP3* overexpression using FoxP3-AAV. For mRNA expression, our data indicated that the downregulation effect of AngII on *FoxP3* was weakened, while the upregulation effect of TP on *FoxP3* persisted in the cells treated with FoxP3-AAV (Figure 6A). Not surprisingly, FoxP3-AAV markedly downregulated *Parkin* mRNA expression. These results implied that both the upregulation effect of AngII and downregulation effect of TP on *Parkin* mRNA expression were completely lost in cells treated with FoxP3-AAV (Figure 6A).

For mitophagy activation, AngII could induce Parkin-mediated mitophagy in cells treated with the negative control-AAV, but this activity was completely lost in cells treated with FoxP3*-*AAV (Figure 6B-C). TP inhibition of Parkin-mediated mitophagy was also lost in the cells treated with FoxP3*-*AAV (Figure 6B-C). Collectively, our data suggested that FoxP3 restricted Parkin-mediated cardiac mitophagy and was essential for the effect of TP.

### FoxP3 deficiency enhanced mitophagy in CR

**Figure 7:**
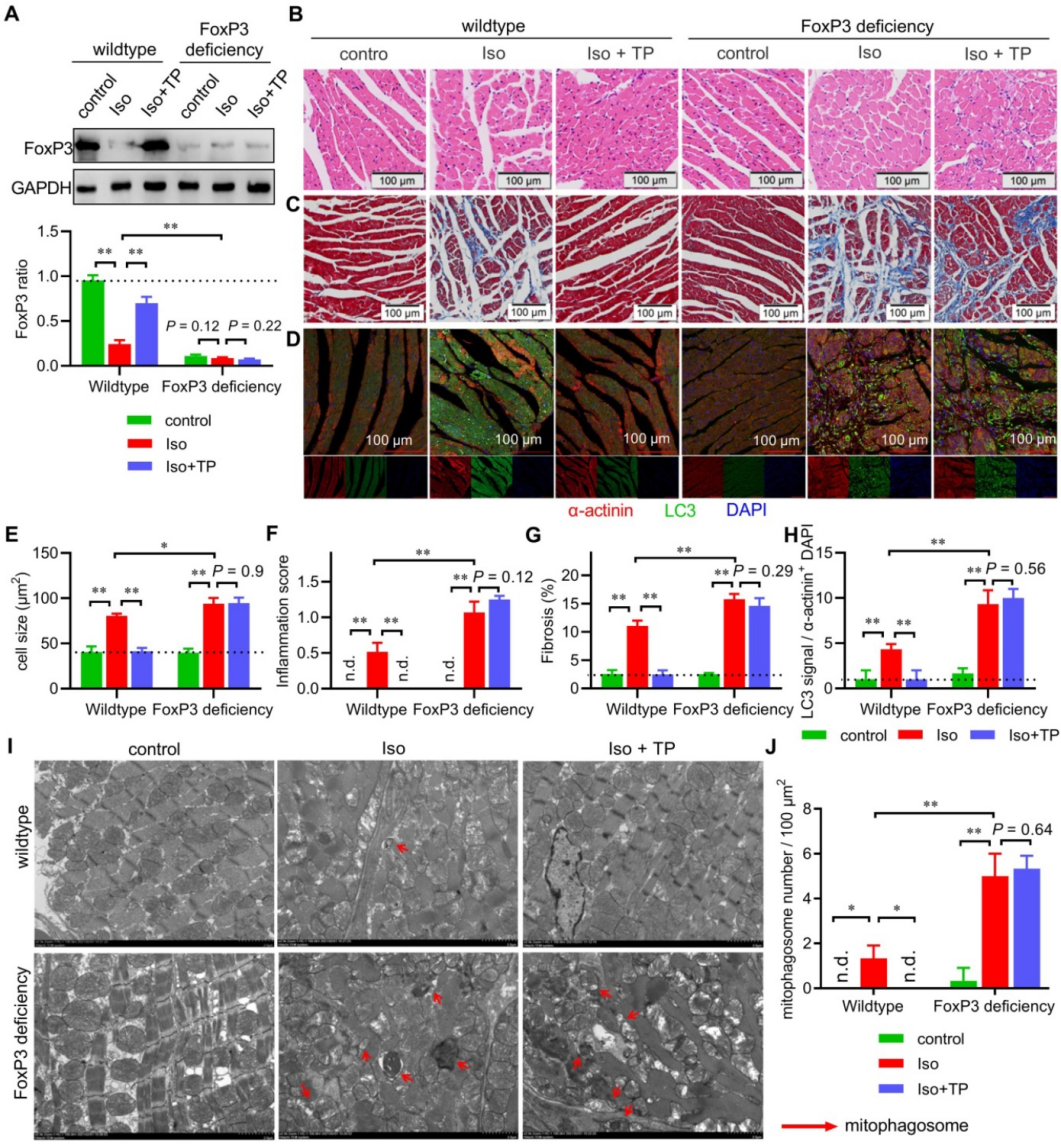
** FoxP3 deficiency enhances mitophagy in cardiac remodeling (CR).** Male FoxP3^DTR^ mice were given an injection of diphtheria toxin (50 μg/kg; i.p.) to deplete cardiomyocytic FoxP3. Then, male FoxP3^DTR^ mice (FoxP3 deficiency) or wild-type C57 mice (wild-type) were treated as shown in Figure 1A. Total lysates or cross-sections of the middle ventricles were used for subsequent assays. (A) Total lysates were used for immunoblotting (IB) with FoxP3 antibody by normalization to GAPDH (n = 3). (B & E-F) HE staining, cell size and inflammation score. (C & G) Masson staining. (D & H) Immunofluorescence (IF) staining for myocardial LC3. The experiments were performed as shown in Figure 2A. (I-J) Transmission electron microscopy (TEM) observations (×2500). The experiments were performed as shown in Figure 3E (n = 3). "n.d." indicates non-detected, ^*^P < 0.05, ^**^P < 0.01.

Our *in vitro* data strongly suggested that FoxP3 inhibited Parkin-mediated cardiac mitophagy. To understand its effect *in vivo*, we used FoxP3^DTR^ mice to further verify the role of FoxP3 in mitophagy during CR. The IB results confirmed that cardiomyocytic FoxP3 dramatically declined in FoxP3-deficient compared with wild-type mice (Figure 7A). In the wild type, cardiomyocytic FoxP3 was downregulated by Iso but was restored by TP treatment, which was similar to the IHC data shown in Figure 1A. However, the downregulation effect of Iso and upregulation effect of TP on cardiomyocytic FoxP3 were completely lost in FoxP3 deficiency, which was similar to the real-time PCR data shown in Figure 5C.

Histological observations of the middle ventricle indicated that Iso induced hypertrophy, fibrosis and inflammatory cell infiltration of the LV, while TP treatment markedly decreased the hypertrophic response (Figure 7B & E), inflammation score (Figure 7B & F) and fibrosis (Figure 7C & G) in wild-type mice. However, Iso showed stronger activity in inducing LV remodeling, and TP completely lost its protective effect in FoxP3-deficient mice. Importantly, IF imaging showed that myocardial autophagy was induced by Iso but inhibited by TP in wild-type mice (Figure 7D & H). In FoxP3-deficient mice, Iso-induced myocardial autophagy was significantly higher than that in wild-type mice, and TP lost its inhibitory effect. Furthermore, the above ventricular specimens were also examined by TEM. In the wild-type group, only a few autophagosomes engulfed with mitochondria were observed in remodeled ventricles (Figure 7I-J). However, many more of these vesicles appeared in remodeled LVs in FoxP3-deficient mice. Not surprisingly, the anti-mitophagy activity of TP persisted in wild-type mice but was lost in FoxP3-deficient mice. Together, these findings suggested that FoxP3 improved CR, inhibited cardiac mitophagy and was required for the activity of TP.

### FoxP3 interacted with the promoter of the *Parkin* gene to downregulate its mRNA expression

**Figure 8:**
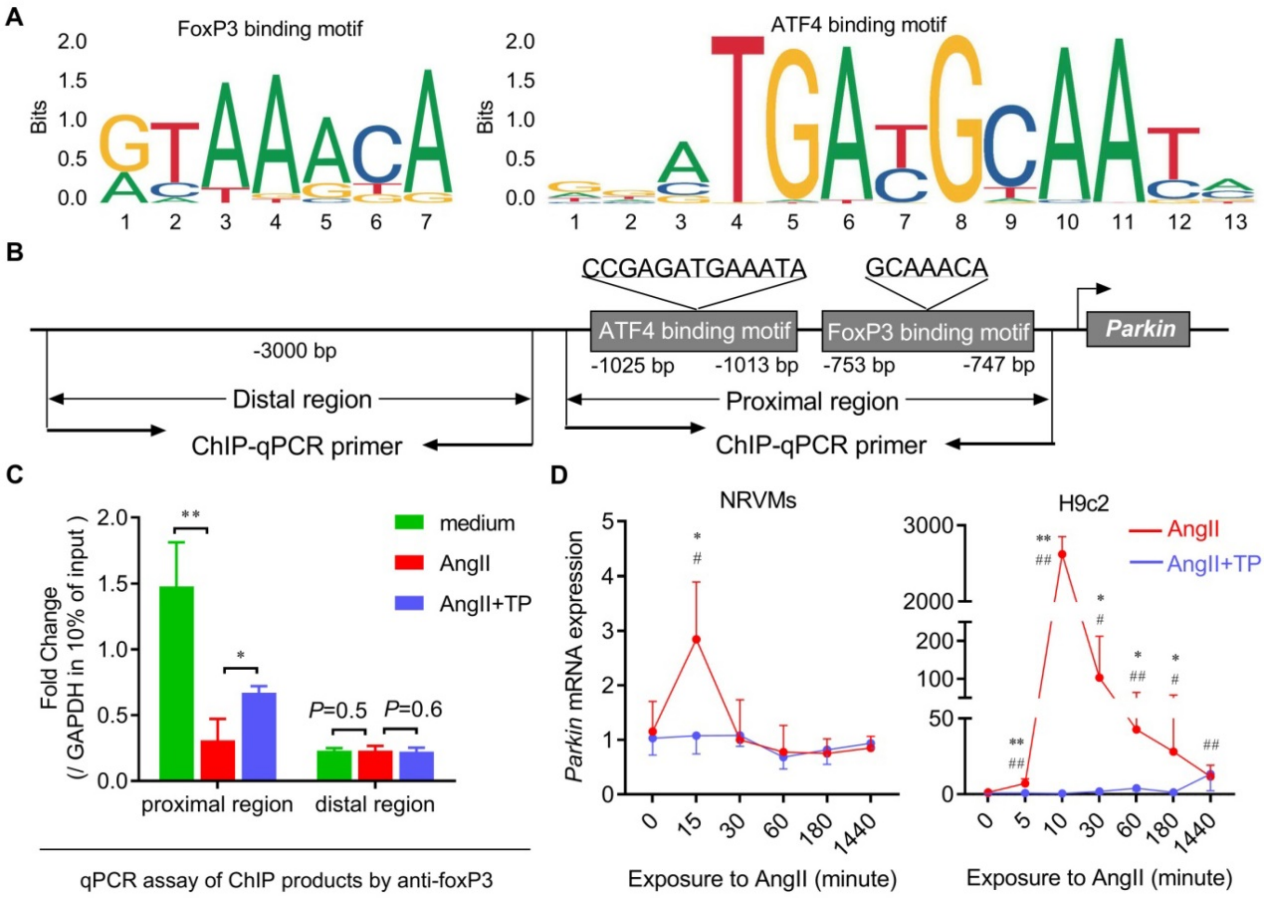
** FoxP3 interacts with the promoter of the* Parkin* gene to downregulate its mRNA expression.** (A) The binding motifs for FoxP3 and ATF4 included in the promoter region of the *Parkin* gene were predicted by JASPAR. (B) A schematic diagram of the loci of primer pairs designed for chromatin immunoprecipitation (ChIP) targeting the distal and proximal regions in the *Parkin* promoter. (C) ChIP-real-time PCR (ChIP-qPCR) assays of the binding of FoxP3 to the promoter of *Parkin* normalized to *gapdh* in the input. The data shown are representative of two independent experiments (n = 3). ^*^P < 0.05, ^**^P < 0.01. (D) H9c2 cells and NRVMs grown in 12-well plates were treated with AngII (1 μM) with or without TP (10 μg/L) for up to 1440 min as indicated, and then cells were harvested for real-time PCR analysis of *Parkin* mRNA expression by normalization to β-actin (n = 3). ^*^P < 0.05, ^**^P < 0.01 vs. 0 h; ^#^*P* < 0.05, ^##^*P* < 0.01 vs. AngII.

Considering its transcriptional activity, we hypothesized that FoxP3 might bind to the promoter of the *Parkin* gene to regulate its mRNA expression. Using the JASPAR online tool (www.jaspar.genereg.net), a FoxP3-binding motif downstream of ATF4 was identified in the promoter region of *Parkin* (Figure 8A-B). Therefore, a ChIP assay was performed to determine whether FoxP3 directly bound to the promoter of *Parkin* in CR. First, we designed a primer pair used for ChIP-qPCR targeting the proximal region containing a FoxP3-binding motif downstream of the ATF4-binding motif, as well as a primer pair targeting the distal region that was used as a negative control (Figure 8B). The ChIP results showed that the binding of FoxP3 to the promoter of *Parkin* was remarkably inhibited by AngII but was partly restored by TP treatment within the proximal region (Figure 8C). No significant binding was detected within the distal region or the IgG pull-down products (data not shown), indicating that our ChIP experiments were specific (Figure 8C). Our results indicated that FoxP3 bound to the promoter of Parkin. Moreover, its decreased binding capacity in the AngII group and increased binding capacity in the treatment group might be related to the altered *Parkin* mRNA expression.

Subsequently, the temporal changes in *Parkin* mRNA expression in cardiomyocytes treated with AngII and/or TP were determined, and the results were consistent with the IB results (shown in Figure 4). In both H9c2 cells and NRVMs, *Parkin* mRNA expression in the AngII group increased rapidly within 10-15 min and then decreased gradually (Figure 8D). Not surprisingly, *Parkin* mRNA expression was downregulated continuously by TP treatment (Figure 8D). Taken together, our data suggested that FoxP3 could interact with a downstream motif of the ATF4-binding motif included in the promoter of *Parkin* and downregulate its mRNA expression. The decreased binding of FoxP3 was related to excessive Parkin-mediated mitophagy in CR, while TP restored this binding to inhibit Parkin-mediated mitophagy.

### FoxP3 bound to nuclear ATF4 to hijack free ATF4 in CR

**Figure 9:**
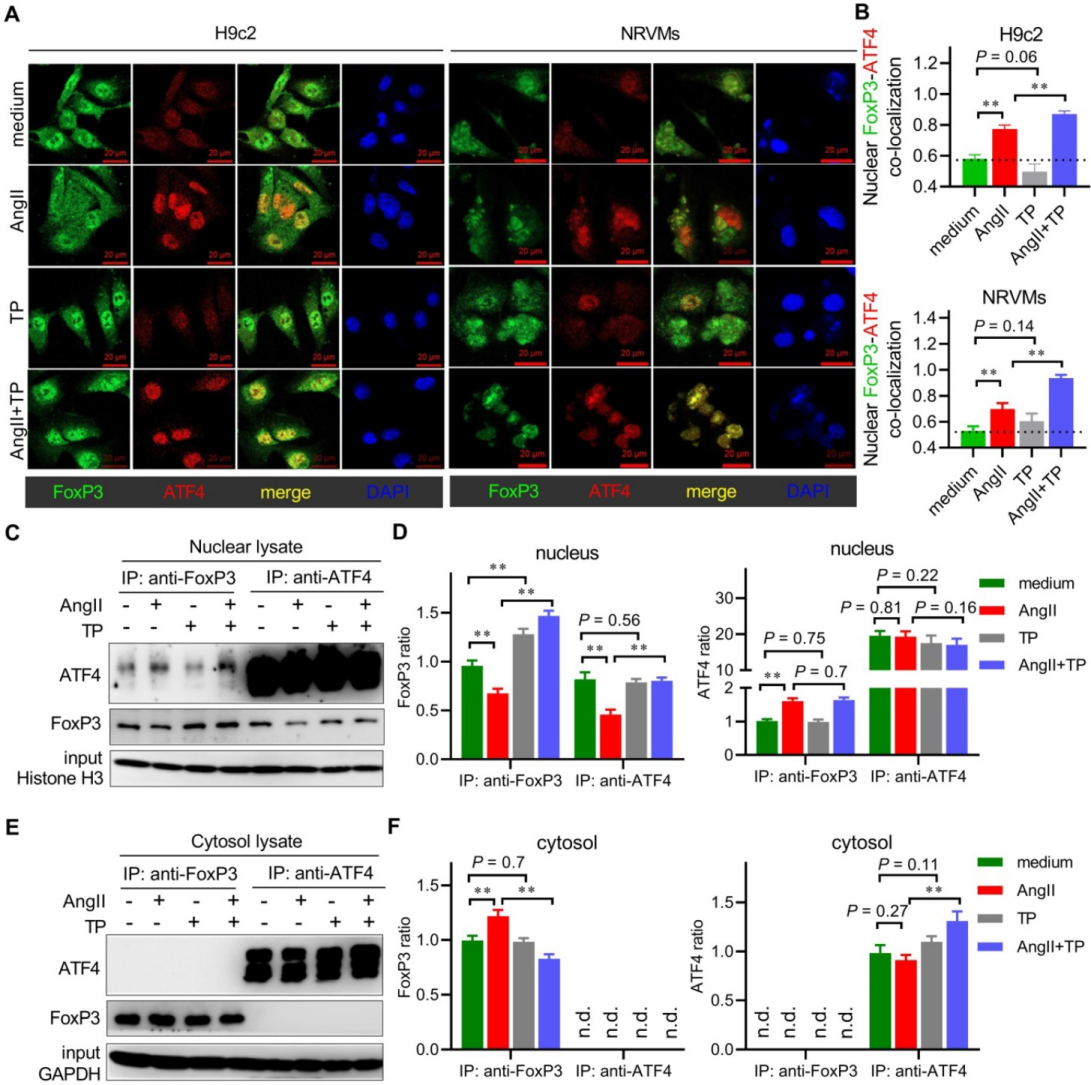
** FoxP3 binds to nuclear ATF4 to hijack free ATF4 in cardiac remodeling (CR).** (A-B) H9c2 cells and NRVMs grown on glass slides were treated with AngII (1 μM) with or without TP (10 μg/L) for 1 h. Then, the cells were fixed, blocked, and probed with antibodies against FoxP3 (green) and ATF4 (red). Confocal imaging and colocalization (yellow) were performed as shown in Figure 3I. (C-D) H9c2 cells grown in 10-cm dishes were treated as in Figure 3B for 1 h. Then, nuclear lysates were subjected to immunoprecipitation (IP) using an antibody against FoxP3, and the associated ATF4 was detected by immunoblotting (IB) or using an antibody against ATF4, and the associated FoxP3 was detected by IB. Protein ratios were calculated by normalization to histone H3 in the input (n = 3). (E-F) Experiments were performed as shown in C-D, and cytoplasmic lysates were used. Protein ratios were calculated by normalization to GAPDH in input (n = 3). ^*^P < 0.05, ^**^P < 0.01.

We also investigated the possibility that FoxP3 might directly interact with ATF4 itself. First, a colocalization experiment was used to observe FoxP3-ATF4 interactions in cardiomyocytes treated with AngII and/or TP. Confocal images showed that ATF4 mainly occurred in the nucleus, resulting in FoxP3-ATF4 colocalization (yellow) only in the nucleus of both H9c2 cells and NRVMs (Figure 9A). Importantly, FoxP3-ATF4 colocalization was increased significantly by AngII stimulation (Figure 9A-B). Although TP treatment could not alter the level of ATF4 signaling, it further increased FoxP3-ATF4 colocalization compared with AngII alone, which suggested that nuclear FoxP3-ATF4 binding was increased by AngII and that TP treatment could further enhance nuclear FoxP3-ATF4 binding.

Subsequently, a co-IP experiment was used to verify the change in FoxP3-ATF4 binding during CR. In the FoxP3 pulldown products from the nuclear lysate, we found that the level of FoxP3 was decreased by AngII but increased by TP, which was in line with the IB results, as shown in Figure 4. Moreover, nuclear FoxP3-associated ATF4 was increased by AngII and was increased more significantly by TP treatment (Figure 9C-D). In the ATF4 pulldown products from the nucleus, the level of ATF4 showed no change in each group, while nuclear ATF4-associated FoxP3 was decreased by AngII and restored by TP treatment (Figure 9C-D). However, the Co-IP results from cytosolic lysates were very different. In the FoxP3 pulldown products, the level of FoxP3 was only weakly increased by AngII, and cytosolic FoxP3-associated ATF4 could not be detected (Figure 9E-F). In the ATF4 pulldown products, the level of ATF4 was only weakly increased by TP treatment, and cytosolic ATF4-associated FoxP3 could not be detected (Figure 9E-F). Collectively, our data supported that FoxP3 bound to nuclear ATF4 to hijack free ATF4, which is considered another mechanism for the negative regulation of Parkin-mediated mitophagy in CR. In addition, the ability of TP to increase this binding might be related to the inhibition of Parkin-mediated mitophagy in CR.

## Discussion

**Figure 10:**
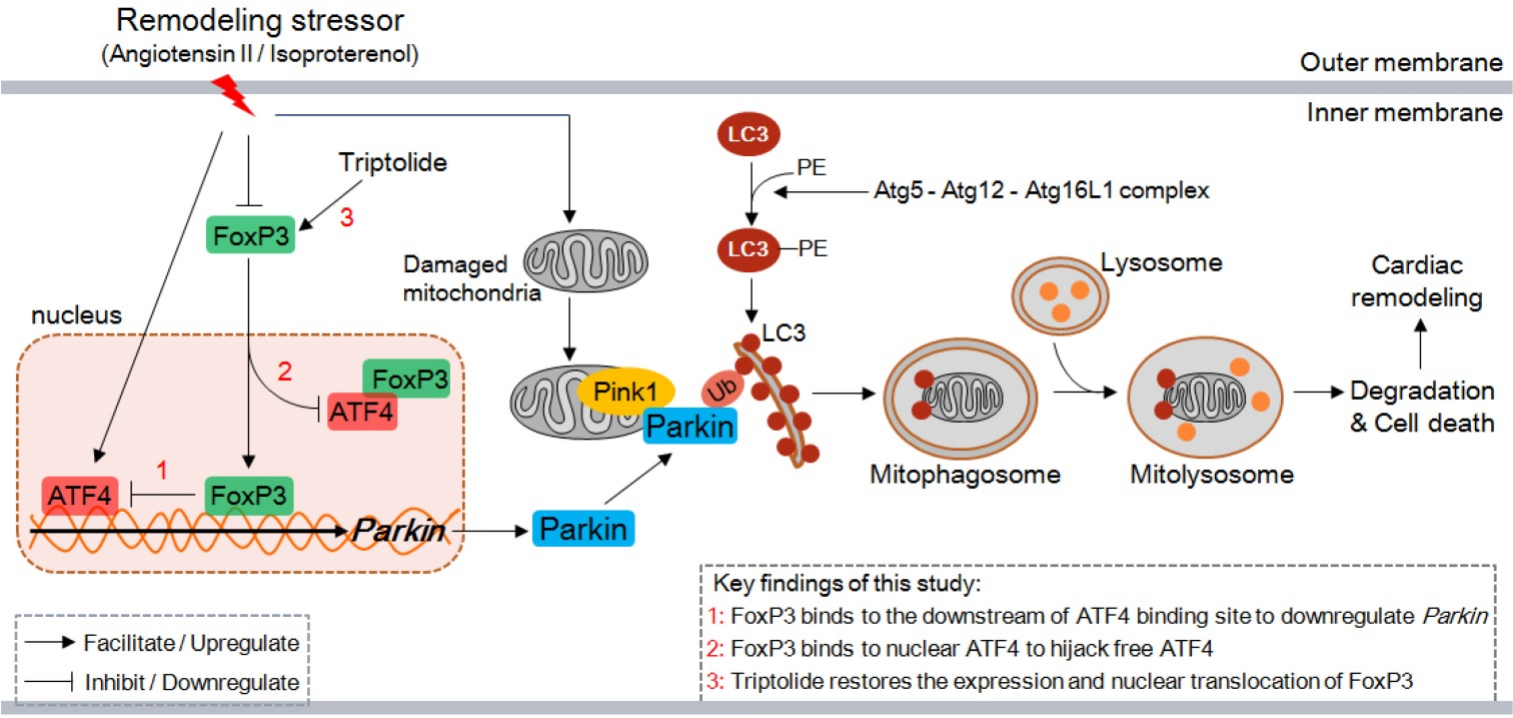
Proposed model for the negative regulation of Parkin-mitophagy by FoxP3 responsible for cardiac remodeling and the effect of triptolide. ATF4, activating transcription factor 4; Atg, autophagy-related protein; FoxP3, Forkhead/winged helix-transcription factor P3; LC3, microtubule-associated protein 1 light chain 3; PE, phosphatidyl ethanolamine; Ub, ubiquitin.

In the present study, we demonstrate several novel findings concerning the role of FoxP3 in CR. First, excessive autophagy occurs early and continuously in the remodeling LV in mice, and the main type is Parkin-mediated myocardial mitophagy. Second, Parkin-mediated mitophagy is enhanced by knocking down or knocking out FoxP3, while it is inhibited by overexpression of FoxP3, indicating that FoxP3 participates in the downregulation of *Parkin*. Third, FoxP3 decreases *Parkin* mRNA expression by interrupting ATF4 activity via two distinct mechanisms, i.e., FoxP3 interacts downstream of the ATF4-binding motif included in the promoter of *Parkin* and binds with nuclear ATF4, thereby hijacking free ATF4 to restrict its transcriptional activity. Moreover, we suggest that the cardioprotective agent TP can restore cardiomyocytic FoxP3 activity to inhibit cardiac mitophagy and attenuate CR (Figure 10). Taken together, cardiomyocytic FoxP3 is involved in the negative regulation of Parkin-mediated mitophagy and contributes to attenuate CR.

As the main functional and structural cell type of the heart, adult cardiomyocytes almost completely lose their capacity for proliferation. Although CR affects all cell types in the heart, changes in cardiomyocytes play a central role 1. Mild stimuli factors, such as hypertension, induce chronic/adaptive CR that is characterized mainly by cardiac hypertrophy; however, it may eventually lead to maladaptive CR under certain conditions. In contrast, strong stimuli, such as MI, acute hemodynamic change, and severe myocarditis, will induce acute/maladaptive CR characterized by sequential alterations of the cardiomyocytes including metabolic imbalance, mitochondrial dysfunction, cell hypertrophy and death, as well as fibrosis and inflammatory cell infiltration in the interstitium, finally leading to heart failure 1. Although the etiologies of these CRs are quite different, they share several pathways in terms of molecular, biochemical and mechanical events and possess a common feature, cardiomyocyte hypertrophy. In this study, we used an Iso-induced mouse model that showed rapid progression of CR, which was more similar to acute/maladaptive CR and conferred more severe clinical performance and consequences than compensatory CR. Iso-stimulated CR induces massive mitochondrial damage, mitophagy and hypertrophy in cardiomyocytes, and significant fibrosis and inflammatory cell infiltration in the interstitium (Figure 1-3).

Prior work in our lab has shown that cardiomyocytic FoxP3 is downregulated and might at least partly participate in CR 10. Here, we reproduced these data and further exhibited the spatiotemporal expression characteristics of cardiomyocytic FoxP3 in CR, finding that the expression and nuclear translocation of FoxP3 were reduced during CR *in vitro* and *in vivo*. To our knowledge, in addition to the literature mentioned in the Introduction, the beneficial effects of FoxP3 expressed in Tregs in experimental CR models have been reported in the past decade 17. An earlier report indicated that Tregs were recruited to the infarcted myocardium to modulate fibroblast phenotype and function in a mouse MI model 18. Another simultaneous report, in contrast, indicated that the functional activity of Tregs was suppressed in mice with MI, while adoptive transfer of Tregs significantly attenuated subsequent CR 19. A recent report also indicated that the Treg subset, which was proinflammatory and antiangiogenic, conferred a critical pathogenic role in promoting pathological LV remodeling in a chronic ischemic heart failure model, while restoration of normal Treg function showed therapeutic potential 20. In summary, FoxP3 could effectively improve the myocardial interstitium and inflammatory microenvironment by controlling the functions of circulating Tregs. In other words, these regulatory effects are indirect in CR. Based on our data on cardiomyocytic FoxP3, it becomes quite attractive to determine whether and how cardiomyocyte-derived FoxP3 is involved in CR. As a well-studied transcription factor, the target genes of FoxP3 in immune cells have been reported, but fewer of them are known within cardiomyocytes.

Combined with several morphologic observations and molecular assessments, it was found that mitophagy, a selective autophagy wherein cells eliminate damaged or dysfunctional mitochondria, was excessively activated in CR. Mitochondria are the most abundant organelles in cardiomyocytes and are responsible for the maintenance of cell function 21. Damaged mitochondria produce many reactive oxygen species (ROS), which damage proteins, membranes, nucleic acids, and adjacent healthy mitochondria and release proapoptotic proteins into the cytosol, finally leading to cell death 21, 22. Therefore, the clearance of damaged mitochondria executed by mitophagy is vital for cell survival and heart function. However, other strong evidence also suggests that extreme mitophagy activation is also related to cardiac injury and subsequent CR 23, 24. In addition, although damaged mitochondria are excessively cleared by increased mitophagy, the remaining mitochondria cannot accommodate the energy demand of cardiomyocytes and eventually result in cell death 6. Our data suggest that excessive mitophagy is involved in CR induced by Iso because mitophagy enhancement (by FoxP3 deficiency) exacerbates CR, while mitophagy inhibition (by FoxP3 overexpression) improves CR. Mitophagy regulation is thought to be a promising strategy to reverse CR; hence, it is valuable to unveil the regulatory mechanism of cardiac mitophagy and to develop corresponding targeted agents.

To date, Pink1 and Parkin are well-studied autophagy adaptors that are involved in cardiac mitophagy 3, 6. Pink1 is recruited to damaged mitochondria to phosphorylate Parkin (an E3 ubiquitin ligase) and thus activate Parkin; the latter links LC3 to initiate mitophagy, namely, the Pink1/Parkin-dependent mitophagy pathway 25. Moreover, Pink1 can also link mitophagy in a Parkin-independent manner. Our data indicate that both *Pink1* and *Parkin* knockdown results in decreased mitophagy in remodeling cardiomyocytes, suggesting that AngII-induced mitophagy indeed occurs through the Pink1-Parkin pathway. However, only Parkin is a determinant of the activity of AngII because *Parkin* knockdown shows a stronger inhibition of LC3-II levels. These findings agree with a viewpoint raised previously in which Pink1 plays a pivotal role in orchestrating mitophagy in maintaining normal cardiomyocyte function under physiological conditions, whereas Parkin is more essential for promoting mitophagy to adapt to acute stimuli of CR 24, 26.

Therefore, we continued to seek for upstream regulatory mechanisms that modulate Parkin-mediated mitophagy in cardiomyocytes. By modulating FoxP3 expression *in vivo* and *in vitro*, we confirmed that FoxP3 played a negative role in Parkin-mediated mitophagy and subsequent CR. Our data indicate that FoxP3 deficiency exacerbates LV remodeling, no matter hypertrophy of the cardiomyocytes or deteriorative fibrosis and inflammation in the interstitium (Figure 7). Interestingly, FoxP3 deficiency shows a more serious effect on interstitial fibrosis and inflammation than on cardiomyocytic hypertrophy. To our knowledge, interstitial fibrosis and inflammation is the repair process after cardiac injury or cell death during CR 1. Therefore, we can deduce that FoxP3 may have an independent role in cardiac fibrosis and myocarditis. Given that cardiomyocytic FoxP3 level decreases during CR; therefore, restoring cardiomyocytic FoxP3 expression is a feasible strategy to regulate cardiac mitophagy to protect CR.

The key finding of our study is that FoxP3 restricts ATF4 activity to downregulate *Parkin* mRNA expression. To date, these findings have not been previously reported. In detail, FoxP3 interrupts ATF4 activity via two distinct mechanisms: first, FoxP3 interacts with its binding motif located downstream of the ATF4-binding motif included in the promoter of *Parkin*, and second, it directly binds with nuclear ATF4, thereby hijacking free ATF4. In this way, we can deduce that decreased FoxP3 in CR may result in insufficient inhibition of ATF4, which leads to extreme and rapid upregulation of Parkin-mitophagy involved in the pathogenesis of CR. Foxp3 can also regulate the transcriptional activity of nuclear factor of activated T cells (NFAT) via a similar mechanism: Foxp3 competitively binds with the NFAT-binding motif to interrupt its transcriptional activity and forms a complex with NFAT to hijack free NFAT 27. Combining these findings and our data, we thought that the upregulation of FoxP3-ATF4 binding in CR was a feedback regulatory mechanism for maintaining cardiomyocyte homeostasis, although FoxP3 was downregulated in CR (as shown in Figure 9). In addition, our findings expand the spectrum of FoxP3-interacting transcription factors and provide more evidence for investigating novel functions of FoxP3 in cardiovascular diseases.

TP is a major compound extracted from *Tripterygium wilfordii*, a traditional Chinese herb that is used as an immunosuppressive and anti-inflammatory agent 28. Some reports have suggested its cardioprotective effect in CR through the modulation of inflammation-related processes 10, 11, 29. Moreover, TP treatment can upregulate Foxp3 expression in CD4^+^ cells 30. TP can also modulate macroautophagy in cancer cells, hepatocytes, cardiomyocytes, and podocytes, although at a toxic dose 31. Combining these findings, we employed TP as a tool to unveil the role of cardiomyocyte FoxP3 in cardiac mitophagy. As expected, our data suggest that TP restores FoxP3 activity to downregulate Parkin-mediated mitophagy in CR, which is involved in its protective effect. To our knowledge, regardless of whether the autophagy activator rapamycin targets molecular target of rapamycin (mTOR) or the autophagy inhibitor 3-MA and LY294002 target phosphatidylinositol 3-hydroxy kinase class III (PI3KC3), it has shown limited clinical value due to maladaptive toxicity 32. TP is used traditionally in the treatment of autoimmune disorders, organ rejection, and cancers; thus, its safety has been confirmed, and the dose of TP in this study is within a superlow range compared with its current usages 33. Therefore, it can be used as a potential FoxP3 activator and mitophagy inhibitor to ameliorate CR in the future.

This study still has certain limitations. Although the relationship between Foxp3, Parkin-mediated mitophagy, and CR is well discussed using *in vivo* experiments with FoxP3-deficient mice and* in vitro* studies using genetic modifications, more studies are needed to confirm the role of cardiomyocytic Foxp3 in the development of Parkin-mitophagy using animals with heart-specific FoxP3 knockout. The interactions of FoxP3 with nuclear ATF4 need to be further investigated using structural biology techniques. In addition, it is also quite attractive to unveil the interactions or crosstalk between cardiomyocytic FoxP3 and FoxP3 from other cell types, such as cardiac fibroblasts, tissue-resident immune cells, and circulatory immune cells, during CR.

In summary, we report that cardiomyocytic FoxP3 is involved in the negative regulation of Parkin-mediated mitophagy in the development of CR by interacting with the FoxP3-binding motif located downstream of the ATF4-binding motif involved in the promoter of *Parkin* and by hijacking free nuclear ATF4. In this way, the transcriptional activity of ATF4 is restricted and subsequently results in decreased *Parkin* mRNA expression, which further provides a cardioprotective agent TP that restores cardiomyocytic FoxP3 activity to inhibit cardiac mitophagy and attenuate CR.

## Figures and Tables

**Table 1:** Primer and siRNA sequences used in this study.

Gene	Forward primer (5'→3')	Reverse primer (5'→3')
*FoxP3*	GGCAGAGGACACTCAATGAAAT	TCTCCACTCGCACAAAGCAC
*Parkin*	TGACACCAGCATCTTCCAGC	TCCAGAGGCATTTGTTTCGT
*β-actin*	GAGACCTTCAACACCCCAGC	ATGTCACGCACGATTTCCC
ChIP-*Parkin* distal region	GTTGTATTTAGAAGTAAGGAAGCATT	TAAACCAAGGTCGTCTACTGAG
ChIP-*Parkin* proximal region	AAGCCTATGACCTCTAAGAACC	TACTGGACTAGCCTACAAGCAC
control siRNA	UUC UCC GAA CGU GUC ACG UTT	ACG UGA CAC GUU CGG AGA ATT
*FoxP3* siRNA1	GGUACACCCAGGAAAGACATT	UGUCUUUCCUGGGUGUACCTT
*FoxP3* siRNA2	GGCAGAGGACACUCAAUGATT	UCAUUGAGUGUCCUCUGCCTT
*FoxP3* siRNA3	GCCCUCACAACCAGCUAUATT	UAUAGCUGGUUGUGAGGGCTT
*Atg5-*siRNA1	UCGAGACGUGUGGUUUGGACGGAUU	AAUCCGUCCAAACCACACGUCUCGA
*Atg5-*siRNA2	GGAUUCCAACGUGCUUUACUCUCUA	UAGAGAGUAAAGCACGUUGGAAUCC
*Pink1-*siRNA1	CCGCAUGGCUUUGGAUGGAGAGUAU	AUACUCUCCAUCCAAAGCCAUGCGG
*Pink1-*siRNA2	CGCAUGGCUUUGGAUGGAGAGUAUG	CAUACUCUCCAUCCAAAGCCAUGCGG
*Parkin-*siRNA1	CCAGCAUCUUCCAGCUCAATT	UUGAGCUGGAAGAUGCUGGTT
*Parkin-*siRNA2	GGAACAACAGAGUAUCGUUTT	AACGAUACUCUGUUGUUCCTT
*Parkin-*siRNA3	GCUCAACGAUCGGCAGUUUTT	AAACUGCCGAUCGUUGAGCTT

## Abbreviations

AAV: adeno associated virus
AngII: angiotensin II
ATF4: activating transcription 4
Atg: autophagy-related protein
BNIP3: BCL2 interacting protein 3
ChIP: chromatin immunoprecipitation
CR: cardiac remodeling
DTR: diphtheria toxin receptor
FoxP3: forkhead/winged helix transcriptional factor P3
IB: immunoblotting
IF: immunofluorescence
IHC: immunohistochemistry
IP: immunoprecipitation
Iso: isoproterenol
LC3: microtubule-associated protein 1 light chain 3
LV: left ventricle
mTOR: molecular target of rapamycin
NIX: BNIP3-like protein
PI3KC3: phosphatidylinositol 3-hydroxy kinase class III
Pink1: PTEN-induced kinase 1
siRNA: small interference RNA
TEM: transmission electron microscopy
TP: triptolide

## References

[1] Cohn JN, Ferrari R, Sharpe N (2000). Cardiac remodeling-concepts and clinical implications: a consensus paper from an international forum on cardiac remodeling. Behalf of an International Forum on Cardiac Remodeling. J Am Coll Cardiol.

[2] Nishida K, Otsu K (2016). Autophagy during cardiac remodeling. J Mol Cell Cardiol.

[3] Zhou H, He L, Xu G, Chen L (2020). Mitophagy in cardiovascular disease. Clin Chim Acta; Int J Clin Chem.

[4] Ikeda S, Zablocki D, Sadoshima J (2021). The role of autophagy in death of cardiomyocytes. J Mol Cell Cardiol.

[5] Yamaguchi O, Murakawa T, Nishida K, Otsu K (2016). Receptor-mediated mitophagy. J Mol Cell Cardiol.

[6] Yang Y, Li T, Li Z, Liu N, Yan Y, Liu B (2020). Role of Mitophagy in Cardiovascular Disease. Aging Dis.

[7] Matsumoto K, Ogawa M, Suzuki J, Hirata Y, Nagai R, Isobe M (2011). Regulatory T lymphocytes attenuate myocardial infarction-induced ventricular remodeling in mice. Int Heart J.

[8] Kvakan H, Kleinewietfeld M, Qadri F, Park JK, Fischer R, Schwarz I (2009). Regulatory T cells ameliorate angiotensin II-induced cardiac damage. Circulation.

[9] Kasal DA, Barhoumi T, Li MW, Yamamoto N, Zdanovich E, Rehman A (2012). T regulatory lymphocytes prevent aldosterone-induced vascular injury. Hypertension.

[10] Ding YY, Li JM, Guo FJ, Liu Y, Tong YF, Pan XC (2016). Triptolide Upregulates Myocardial Forkhead Helix Transcription Factor p3 Expression and Attenuates Cardiac Hypertrophy. Front Pharmacol.

[11] Pan XC, Liu Y, Cen YY, Xiong YL, Li JM, Ding YY (2019). Dual Role of Triptolide in Interrupting the NLRP3 Inflammasome Pathway to Attenuate Cardiac Fibrosis. Int J Mol Sci.

[12] Wilson HM, Cheyne L, Brown PAJ, Kerr K, Hannah A, Srinivasan J (2018). Characterization of the Myocardial Inflammatory Response in Acute Stress-Induced (Takotsubo) Cardiomyopathy. Jacc-Basic Transl Sci.

[13] Jiang B, Zhang B, Liang P, Song J, Deng H, Tu Z (2010). Nucleolin/C23 mediates the antiapoptotic effect of heat shock protein 70 during oxidative stress. FEBS J.

[14] Livak KJ, Schmittgen TD (2001). Analysis of relative gene expression data using real-time quantitative PCR and the 2(-Delta Delta C(T)) Method. Methods.

[15] Yan J, Yan JY, Wang YX, Ling YN, Song XD, Wang SY (2019). Spermidine-enhanced autophagic flux improves cardiac dysfunction following myocardial infarction by targeting the AMPK/mTOR signalling pathway. Br J Pharmacol.

[16] Sciarretta S, Maejima Y, Zablocki D, Sadoshima J (2018). The Role of Autophagy in the Heart. Annu Rev Physiol.

[17] Meng X, Yang J, Dong M, Zhang K, Tu E, Gao Q (2016). Regulatory T cells in cardiovascular diseases. Nat Rev Cardiol.

[18] Saxena A, Dobaczewski M, Rai V, Haque Z, Chen W, Li N (2014). Regulatory T cells are recruited in the infarcted mouse myocardium and may modulate fibroblast phenotype and function. Am J Physiol-Heart C.

[19] Sharir R, Semo J, Shimoni S, Ben-Mordechai T, Landa-Rouben N, Maysel-Auslender S (2014). Experimental myocardial infarction induces altered regulatory T cell hemostasis, and adoptive transfer attenuates subsequent remodeling. PloS one.

[20] Bansal SS, Ismahil MA, Goel M, Zhou G, Rokosh G, Hamid T (2019). Dysfunctional and Proinflammatory Regulatory T-Lymphocytes Are Essential for Adverse Cardiac Remodeling in Ischemic Cardiomyopathy. Circulation.

[21] Lu YP, Ding WW, Wang B, Wang L, Kan HW, Wang XT (2020). Positive regulation of human PINK1 and Parkin gene expression by nuclear respiratory factor 1. Mitochondrion.

[22] Bravo-San Pedro JM, Kroemer G, Galluzzi L (2017). Autophagy and Mitophagy in Cardiovascular Disease. Circ Res.

[23] Nah J, Fernandez AF, Kitsis RN, Levine B, Sadoshima J (2016). Does Autophagy Mediate Cardiac Myocyte Death During Stress?. Circ Res.

[24] Li YZ, Wu XD, Liu XH, Li PF (2019). Mitophagy imbalance in cardiomyocyte ischaemia/reperfusion injury. Acta Physiol.

[25] Vasquez-Trincado C, Garcia-Carvajal I, Pennanen C, Parra V, Hill JA, Rothermel BA (2016). Mitochondrial dynamics, mitophagy and cardiovascular disease. J Physiol.

[26] Kubli DA, Zhang X, Lee Y, Hanna RA, Quinsay MN, Nguyen CK (2013). Parkin protein deficiency exacerbates cardiac injury and reduces survival following myocardial infarction. J Biol Chem.

[27] Bandukwala HS, Wu Y, Feuerer M, Chen Y, Barboza B, Ghosh S (2011). Structure of a domain-swapped FOXP3 dimer on DNA and its function in regulatory T cells. Immunity.

[28] Tong L, Zhao Q, Datan E, Lin GQ, Minn I, Pomper MG (2021). Triptolide: reflections on two decades of research and prospects for the future. Nat Prod Rep.

[29] Zhang Z, Qu X, Ni Y, Zhang K, Dong Z, Yan X (2013). Triptolide protects rat heart against pressure overload-induced cardiac fibrosis. Int J Cardiol.

[30] Zhang GT, Liu Y, Guo HQ, Sun ZY, Zhou YH (2009). Triptolide promotes generation of FoxP3+T regulatory cells in rats. J Ethnopharmacol.

[31] Wei YM, Wang YH, Xue HQ, Luan ZH, Liu BW, Ren JH (2019). Triptolide, A Potential Autophagy Modulator. Chin J Integ Med.

[32] Zhang K, Zhu S, Li J, Jiang T, Feng L, Pei J (2021). Targeting autophagy using small-molecule compounds to improve potential therapy of Parkinson's disease. Acta Pharm Sin B.

[33] Li XJ, Jiang ZZ, Zhang LY (2014). Triptolide: progress on research in pharmacodynamics and toxicology. J Ethnopharmacol.

